# Unraveling the Morphological Variation of *Triatoma infestans* in the Peridomestic Habitats of Chuquisaca Bolivia: A Geometric Morphometric Approach

**DOI:** 10.3390/insects12020185

**Published:** 2021-02-22

**Authors:** Carolina Vilaseca, Marco A. Méndez, Carlos F. Pinto, Darija Lemic, Hugo A. Benítez

**Affiliations:** 1 Laboratorio de Ecología Química, Universidad Mayor Real y Pontificia San Francisco Xavier de Chuquisaca, Sucre, Bolivia; vilaseca_c@yahoo.com.ar (C.V.); leqcepi@gmail.com (C.F.P.); 2 Laboratorio de Genética y Evolución, Facultad de Ciencias, Instituto de Ecología y Biodiversidad, Universidad de Chile, Santiago 6640022, Chile; mmendez@uchile.cl; 3 Department of Agricultural Zoology, Faculty of Agriculture, University of Zagreb, Svetošimunska 25, 10000 Zagreb, Croatia; dlemic@agr.hr; 4 Laboratorio de Ecología y Morfometría Evolutiva, Centro de Investigación de Estudios Avanzados del Maule, Universidad Católica del Maule, Talca 3466706, Chile

**Keywords:** *Triatoma infestans*, sexual dimorphism, shape plasticity, geometric morphometric, Chagas disease

## Abstract

**Simple Summary:**

*Triatoma infestans* is the main vector of the Chagas disease transmission and has been for years one of the main sanitary problems in Bolivia, particularly for the movement between isolated population to the urban areas. In the following research, we analyze the pattern of biological adaptation of this vector species from two different areas in Bolivia (areas of the Bolivian Chaco with the inter-Andean valleys). Using advanced geometric morphometric tools, it was possible to unravel *T. infestans* morphological variation and understand the biological adaptation of this important insect species.

**Abstract:**

Morphometrics has been used on Triatomines, a well-known phenotypically variable insect, to understand the process of morphological plasticity and infer the changes of this phenomenon. The following research was carried out in two regions of the inter-Andean valleys and two Chaco regions of Chuquisaca-Bolivia. *Triatoma infestans* adults were collected from the peridomestic (pens and chicken coops) along a geographic gradient in order to evaluate the morphological differentiation between groups and their pattern of sexual shape dimorphism. Geometric morphometric methods were applied on the wings and heads of *T. infestans*. The main findings include that we proved sexual dimorphism in heads and wings, determined the impact of environmental factors on size and shape and validated the impact of nutrition on head shape variation. These results show that geometric morphometric procedures can be used to provide key insight into the biological adaptation of *T. infestans* on different biotic (nutrition) and abiotic (environment) conditions, which could serve in understanding and evaluating infestation processes and further vector control programs.

## 1. Introduction

In Bolivia, *Triatoma infestans* (Klug) Hemiptera Reduviidae is the main vector of *Trypanosoma cruzi*, a parasite that causes Chagas disease. *T. infestans* is a synanthropic insect it is found in seven countries in Latin America [1]. In Bolivia, *T. infestans* is not only limited to the intradomiciliary and peridomestic habitat, but in some localities of the inter-Andean valleys and the Chaco, there are sylvatic foci [1,2,3]; however, in Chuquisca no sylvatic foci outbreaks have been identified yet [4]. Environmental characteristics, such as temperature and relative humidity, as well as the structure of the pens and chicken coops, are important factors to understand the morphological characteristics of the insect.

Chuquisaca is a region where the Chagas disease has been endemic in Bolivia; programs for diagnosis and treatment of the disease have been implemented along with the application of chemicals like insecticides in houses and peridomestic habitats, such as pens, chicken coops and warehouses [5]. According to reports from the Chagas Program, the intradomiciliary infestation level in the inter-Andean zone has decreased to less than 3%, but in the Chaco region, it is still high, more than 7% [5]. In the rural peridomestic environment, infestation rates for *T. infestans* are still high in both regions, more than 14%, despite spraying insecticides, with continuous re-infestation processes [5]. Morphometric analyses in *T. infestans* have studied the differentiation between domestic and sylvatic specimens in Bolivia [2,3,6,7,8,9,10].

In the last thirty years, studies that analyze the relationship between wild (sylvatic) and domestic *T. infestans* populations have been performed using linear morphometrics of multiple traits. Recently, geometric morphometrics with the aim to understand patterns of the origins of morphological variation and the level of Chagas infection related to the host and environmental conditions [7,11,12].

Nevertheless, intra and interspecific patterns of sexual dimorphism related to environmental conditions are less studied. Sexual dimorphism is a topic of interest in parasitological studies, particularly using vector species since the differentiation between sexes is often not obvious, or the specimens are very small; thus, finding discriminating characters allows easy determination of sexes [11]. Sexual dimorphism differences in morphological traits are a common phenomenon in insects, and their most conspicuous aspect is the size and ultimately shape [13,14,15,16]. Investigating the pattern of morphological adaptation has been an essential element in comparative biology and invasion biology, and particularly in the study of organismal diversification and evolutionary innovation [17,18,19].

The subfamily Triatominae is well-known as a highly plastic subfamily of insects, where morphometric studies have been used to understand the process of plasticity and sexual dimorphism and infer the changes of these phenomena [20,21]. *Triatoma infestans* (Hemiptera: Reduviidae) is an insect that presents a high level of morphological variation; such variation was described by Dujardin et al. [20] as phenotypic plasticity, an important process to increase or decrease in size in response to short-term environmental variation, while shape variation has a genetic component [22].

Hernández et al. [23] found a relationship between nutritional status and head sexual size dimorphism in triatomines from chicken coops and goat pens studied in their natural environment. Dujardin et al. [24] reported a reduction of sexual dimorphism in head measurements of *T. infestans* populations raised in a laboratory, in similar conditions to an intradomiciliary environment, compared to sylvatic populations, due to the effects of high population density and food competition, hence, females would be smaller than sylvatic specimens because they have higher nutritional requirements.

The aim of this research is to unravel the pattern of morphological variation of *Triatoma infestans* between two contrasting environments and evaluate the presence of sexual shape and size dimorphism in the peridomestic habitat along a geographic gradient in Bolivia.

## 2. Materials and Methods

### 2.1. Study Area

**Figure 1:**
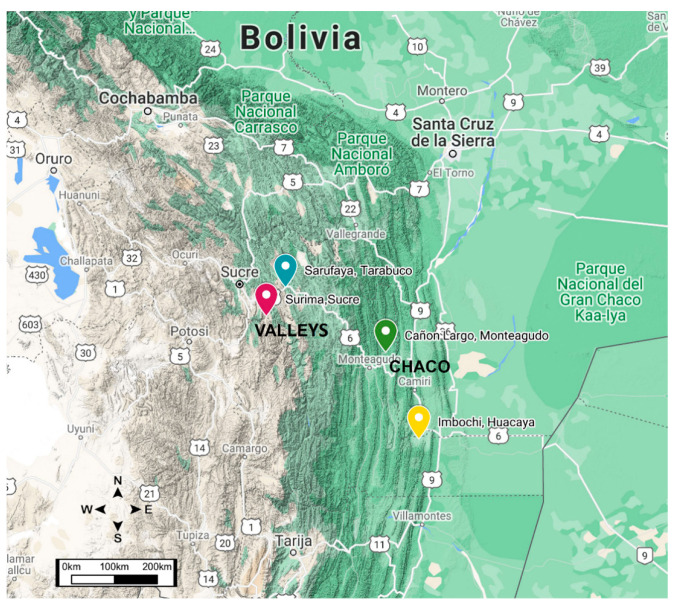
Coordinate information from the Military Institute of Geography of the four locations where *Triatoma infestans* populations were sampled in Chuquisaca.

The study was conducted in four geographical regions of Chuquisaca-Bolivia: two locations in the inter-Andean valleys: Tarabuco/Sarufaya, high valleys (Lat. 19°10′ S Long. 64°54′ O) and Sucre/Surima, low valleys (Lat. 19°29′ S Long. 65°18′ O). Two in the Chaco region, the first one located in the wet Chaco Monteagudo/Cañón Largo (Lat. 19°48′ S Long. 63°57′ O), and the other in the dry Chaco Huacaya/Imbochi (Lat. 20°37′ S Long. 63°10′ O) (Figure 1). The inter-Andean valleys, in the mountainous relief, are in the way of the humid air coming from the east, causing abundant rains. The high inter-Andean valleys are at more than 2900 m above sea level, temperatures are around 17 °C, humidity is 40% per year approximately, and they have high plateau characteristics. Low inter-Andean valleys have a humidity of approximately 50%, the temperature is around 24 °C per year, and they are below 2900 m above sea level [25].

The wet Chaco is also found in the Tucumano–Boliviano region; it is a region of lower mountains, the height of the mountains does not exceed 2600 m above sea level, at the base of the mountains, the altitude is 900 m above sea level. It has a warm and humid climate; the temperature is around 28 °C per year and humidity is close to 50% [25].

The dry Chaco region is located at the east of the Eastern Mountain Range, a region of flat arid lands, related to Paraguay and Northern Argentina, which has a warm climate with annual temperatures above 30 °C, and low humidity, around 20%, it is denominated the Bolivian Boreal Chaco [25].

### 2.2. Insect Sampling and Preparation

A total of 110 adults of *Triatoma infestans* were examined, 57 females and 53 males, distributed as follows: Tarabuco/Sarufaya (TS) 11; 11, Sucre/Surima (SS) 22; 18, Huacaya/Imbochi (HI) 13; 12, Monteagudo/Cañon Largo (MC) 11; 12 females and males, respectively. All were collected in peridomestic locations (pens and chicken coops). We used a gripper to collect the insects, and each insect was placed in a plastic container. The collection was between July and September 2018. The collection of insects was carried out for three weeks per location.

Adult insects were introduced in a plastic container. At least 11 insects of the same sex were collected per location and were preserved in alcohol (96%) for further analyses. In the laboratory, wings were mounted in slides with Euparal ^®^ for the analysis using the right and left wings in all cases. Each head was excised at the collar and mounted on a pin attached to a metal support. All wings and heads were photographed and measured with a Celestron handheld digital microscope pro 5MP.

### 2.3. Morphometric Analysis

**Figure 2:**
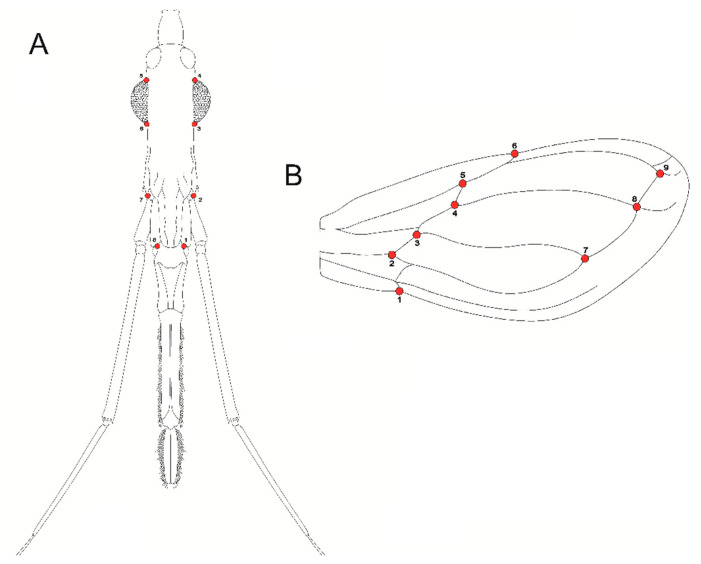
Graphical representation of the landmark positions in head and wing of *Triatoma infestans*. (**A**) Head dorsal view; (**B**) wing view.

Eight landmarks were selected for dorsal views of the head, and nine landmarks of the wings (Figure 2) and digitized using the software TpsDig2 V.231 [26]. For all digitized individuals, the shape information was extracted using a Procrustes superimposition analysis, which is a procedure that removes the information of size, position, and orientation to standardize each specimen according to centroid size [27].

The measurement error (ME) was calculated using a Procrustes ANOVA in order to detect digitizing errors in morphometric data. For this procedure, the original dataset was compared with a control of repeated measures, and the values of the mean squares (MS) of the individual values were compared with the error (dataset of the repeated measurement) [28,29].

To characterize the head and wing shape variation, a principal component analysis (PCA) was carried out based on the covariance matrix of shape. Canonical variate analysis (CVA) methods were used to amplify the shape variation and visualize the sexual shape dimorphism between *T. infestans* populations [30,31,32]. Mahalanobis and Procrustes morphological distances were calculated and reported with their respective *p*-values after a permutation test (10,000 runs). Multivariate regression of shape (dependent variable) on centroid size (independent variable) was performed to analyze if the size has an influence on the shape distribution (allometric effect) of *T. infestans* populations of Inter-Andean valleys and Chaco. All the analyses were performed using the software MorphoJ V.1.06 [29] and the R package Momocs [33].

## 3. Results

The Procrustes ANOVA for assessing the measurement error of head shape showed that the mean square for individual variation exceeded the measurement error: MS error: 0.0000354342 < MS individual: 0.0001282749. The measurement error in the wings showed that the mean square for individual variation exceeded the measurement error (MS error: 0.000099151 < MS individual: 0.0010029078).

**Figure 3:**
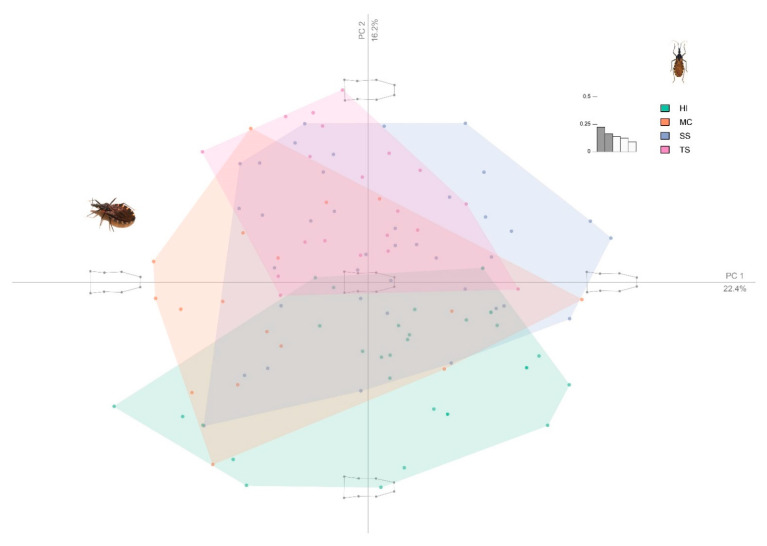
PCA of the *Triatoma infestans* head dorsal view in four populations. Color code: green: Huacaya/Imbochi (HI); orange: Monteagudo/Cañon Largo (MC); blue: Sucre/Surima (SS), and pink: Tarabuco/Sarufaya (TS).

**Figure 4:**
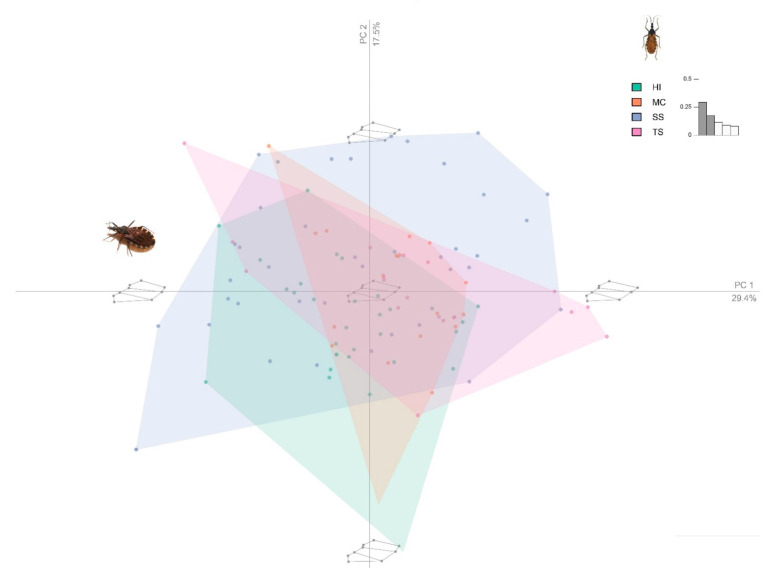
Principal component analysis (PCA) of the *Triatoma infestans* wing view in four populations. Color code: green: Huacaya/Imbochi (HI); orange: Monteagudo/Cañon Largo (MC); blue: Sucre/Surima SS), and pink: Tarabuco/Sarufaya (TS).

**Figure 5:**
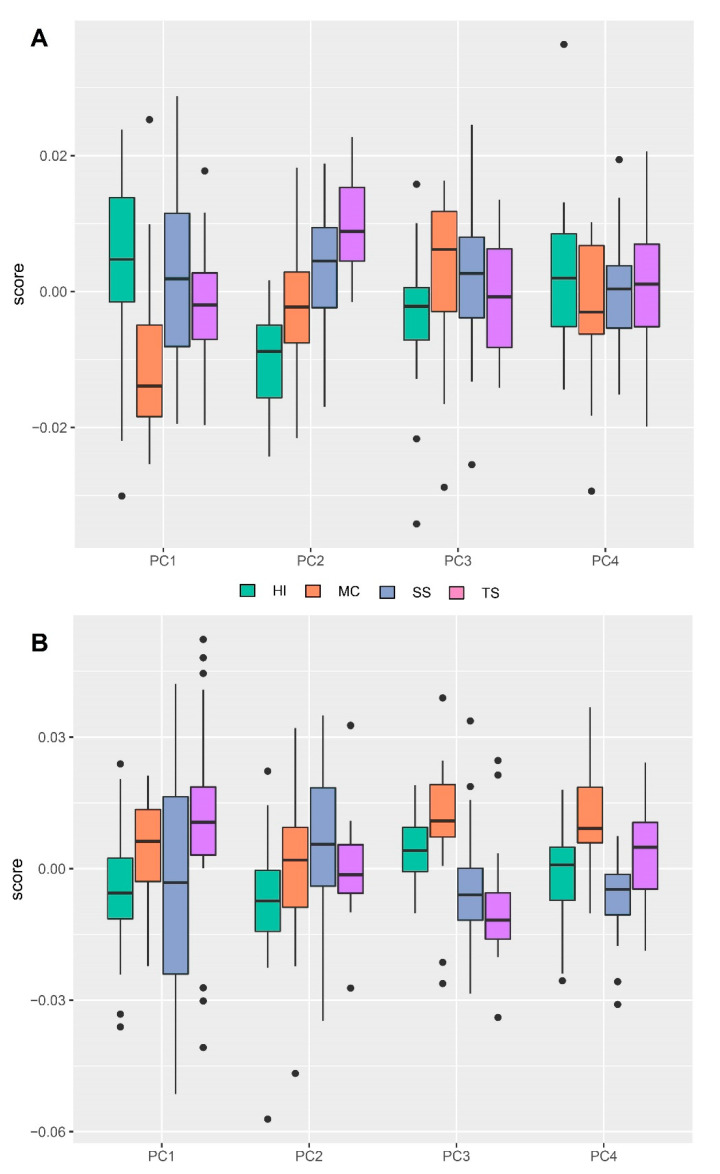
Box Plot of PCA scores for different populations of *T. infestans*. (**A**) Head view; (**B**) wing view. Green: Huacaya/Imbochi (HI); orange: Monteagudo/Cañon Largo (MC); blue: Sucre/Surima (SS), and purple: Tarabuco/Sarufaya (TS).

Principal component analysis showed that the first three PCs accounted for 52.882% of the head shape variation (PC1:22.440%, PC2:16.373%, PC3:14.069%). The PCA of the wings view showed that the first three PCs accounted for 61.055% (PC1:33.499%, PC2:16.613%, PC3:10.743%). In order to localize the shape variation, the average shape was extracted for the two localities of Inter-Andean valleys (Sucre/Surima and Tarabuco/Sarufaya) and two localities of Chaco (Huacaya/Imbochi and Monteagudo/Cañon Largo). The dorsal head view showed that individuals of *T. infestans* from HI were clearly different from the TS, but with few superpositions of individuals with MC and SS and the wing view, the superposition of individuals was more evident for all populations. (Figure 3, Figure 4 and Figure 5).

**Figure 6:**
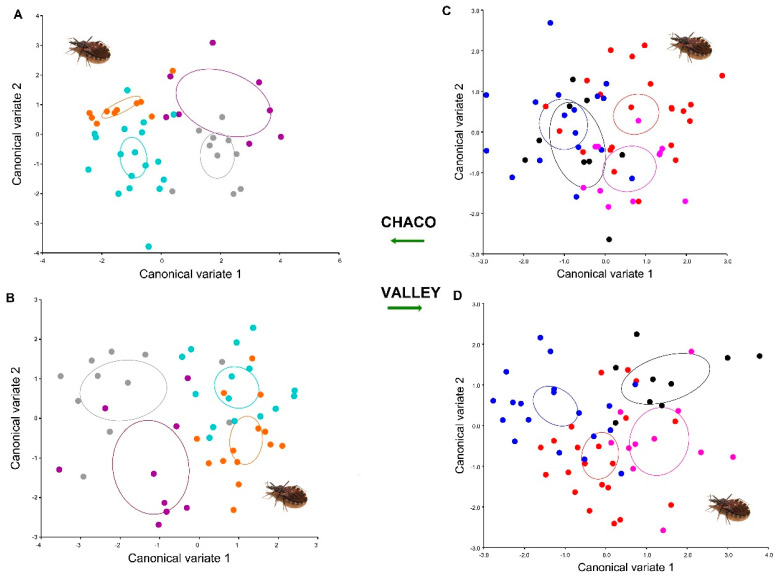
Scatterplot of the canonical variate analysis of *Triatoma infestans* populations of Chaco and Inter-Andean valley (**A**) Canonical variate analysis (CVA) of head dorsal view Huacaya/Imbochi and Monteagudo/Cañon Largo; (**B**) CVA of wing view Huacaya/Imbochi and Monteagudo/Cañon Largo; (**C**) CVA of head dorsal view Tarabuco/Sarufaya and Sucre/Surima; (**D**) CVA of wing view Tarabuco/Sarufaya and Sucre/Surima. Orange: female/Huacaya-Imbochi; light blue: male/Huacaya-Imbochi; gray: female/Monteagudo-Cañón Largo; purple: male/Monteagudo Cañón Largo; red: female/Sucre-Surima: blue; male/Sucre-Surima; pink: female/Tarabuco-Sarufaya; black: male/ Tarabuco Sarufaya.

The scatterplot of CVA shows differentiation between females and males (sexual shape dimorphism) in heads and wings of *T. infestans* populations of Chaco and Inter-Andean valley (Figure 6).

After extracting Mahalanobis and Procrustes distances (permutations 10,000 runs), *T. infestans* did not show sexual shape dimorphism using Procrustes distances for both structures and also for Mahalanobis was not evident in the head of the inter-Andean valleys. According to the relationship between the Mahalanobis Distance (*p* < 0.0001), *T. infestans* populations of Chaco showed sexual shape dimorphism in the head, but not for wings, and, in addition, *T. infestans* populations of inter-Andean valleys presented dimorphism in wings (Table 1).

**Figure 7:**
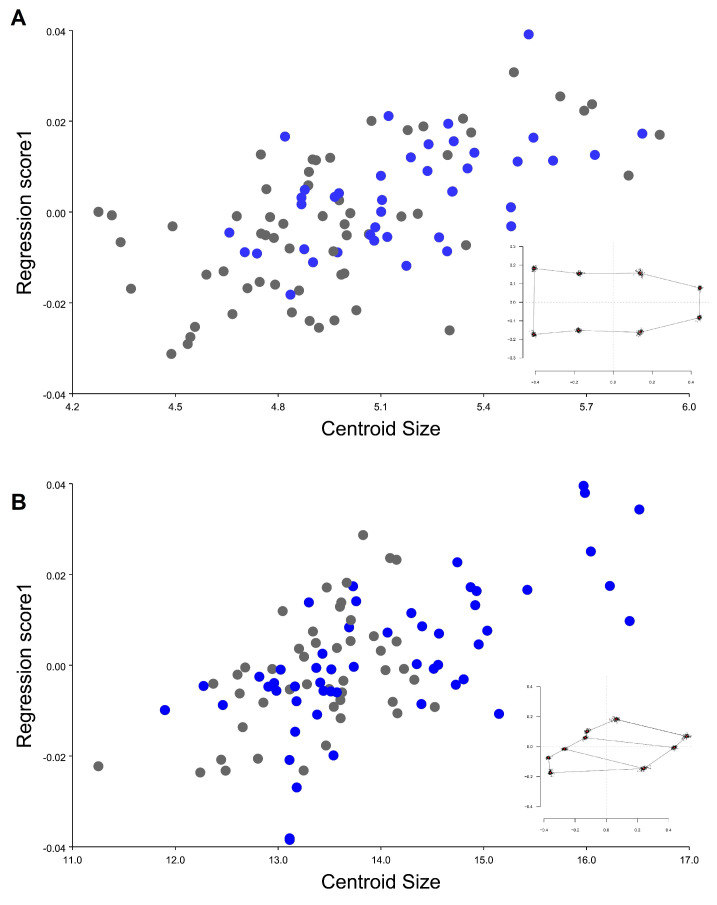
Multivariate regression analysis of *T. infestans* populations, y-axis corresponds to shape (regression scores 1) and the x-axis to size (centroid size). Blue represents the individuals from the inter-Andean valley and gray individuals from Chaco. (**A**) head dorsal view, (**B**) wing view.

**Figure 8:**
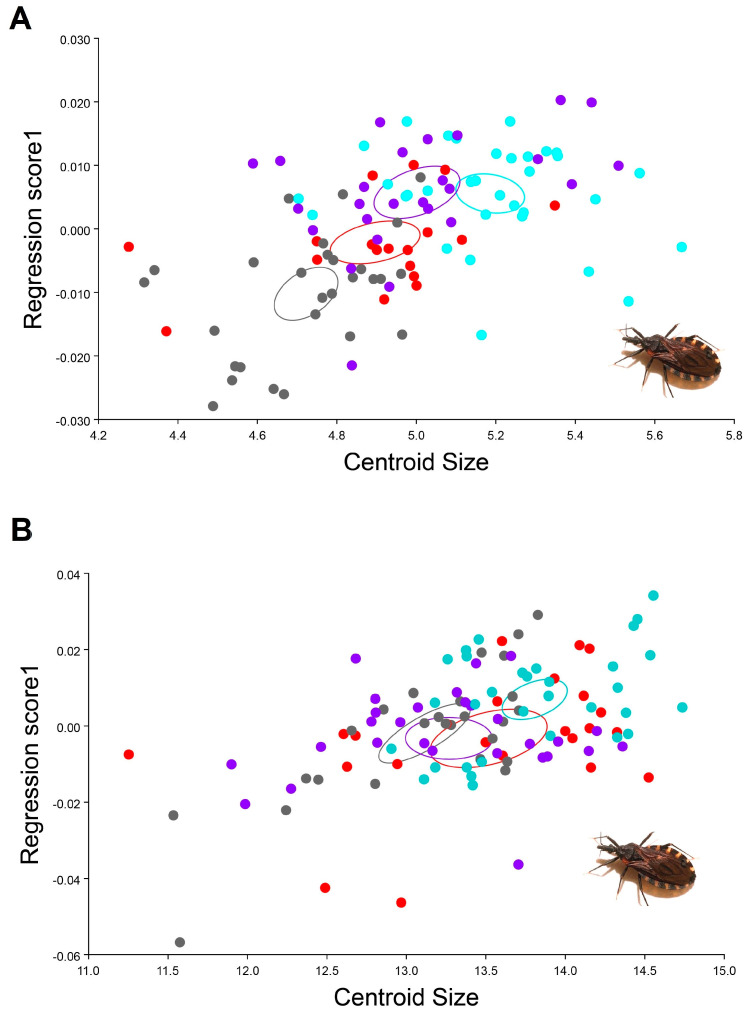
Multivariate regression analysis of *T. infestans* populations, y-axis corresponds to shape and x-axis to size (centroid size). Purple: male Andean valley, gray: male Chaco, light blue: female Andean valley and red: female Chaco. (**A**) dorsal head view, (**B**) wing view.

The multivariate regression showed that, although the allometric percentage was lower, the influence of size was noticeable in the different traits evaluated, where shape variation showed influence by allometry in head and wings; dorsal head view 3.73242% *p*-value 0.0001 and wings view 3.1994% *p*-value < 0.0001. It is possible to identify that *T. infestans* from the Inter-Andean valley are bigger than the specimens from Chaco (see set of gray points at the left of Figure 7A and set of blue points at the right of Figure 7B. (Figure 7). When the analysis is separated between the four analyzed populations, a clear sexual size dimorphism was also observed were males from inter-Andean valleys, and Chaco was smaller in size for both of the traits compared to the females (Figure 8).

## 4. Discussion

This study analyzed the morphological plasticity and sexual shape dimorphism of *T. infestans* in two geographical environments, the inter-Andean valleys and Chaco and found the following results: (A) sexual dimorphism in heads and wings; (B) significant effect of environmental factors on size and shape; (C) impact of nutrition on head shape variation.

(A) In Bolivia, sylvatic-Andean *T. infestans* inhabits rock piles and feeds on animals living in burrows. Morphologically it has a yellow connexivum, similar to the intradomiciliary variety. At the same time, the specimens from boreal Chaco that live in trees and feed on birds have a dark connexivum [34,35,36]. Males often made their movements by flying and prefer peridomestic and intradomicile environments; conversely, females have limited dispersal capabilities, they remain in a single habitat, and they do not discriminate their food source [21]. This research confirmed a significant morphological variation in the head and wings of *T. infestans*. In sylvatic environments, investigated populations of *T. infestans* showed high levels of sex-based dimorphism. Results showed that females are larger than males (discussed in Djuradin et al. [9]). In species like insects, a sexual dimorphism observed in smaller size and shape of males is often revealed in many species of Diptera, Lepidoptera, Hymenoptera and Coleoptera [37,38,39,40,41,42,43]. After further analyses, this survey found that sexual dimorphism depends on geographic region. *T. infestans* populations from Chaco had sexual shape dimorphism in the head but were not observed for wings, while *T. infestans* populations of inter-Andean valleys had sexual dimorphism in wings. According to Fairbairn [44] and Cox et al. [45], sexual dimorphism may be the result of ecological and reproductive pressure. Sexual dimorphism in triatomines can be related to feeding habits and population density [24,46,47]. In the Bolivian Chaco (Huacaya/Imbochi and Monteagudo/Cañon Largo), *T. infestans* were found in the intra- and peridomestic habitat, with increased population density compared to populations from the valleys. High population density may determine an intraspecific competition for food, consequently having males with smaller heads than females [48]. As discussed in Mikac et al. [49] for coleopteran species and in Lemic et al. [42] for dipteran species, it is thought that bigger wings are probably more aerodynamic and may also be useful for mated females that are known to engage in migratory flights. Considering presented results and based on literature review [43,49,50,51,52], this study provides opposite morphological evidence than shown in Hernández et al. [21] that the migration in *T. infestans* can be attributed to the females of this species.

(B) The insect size and shape were influenced by environmental factors. The geography of inter-Andean valleys featuring mountains, temperatures between 17 and 24 °C and humidity above 40%, and the structure of peridomestic environments, such as chicken coops and pens, built with earth blocks that make them dark and wet, are unfavorable factors for the development of *T. infestans* microcolonies. In these unfavorable environmental conditions, females have the priority in feeding, which has a direct influence on their bigger wing size and shape [53,54,55]. *T. infestans* fly in the warmer months when temperatures get close to 30 °C. At temperatures below 20 °C, *T. infestans* do not fly; therefore, re-infestation of peridomicile to intradomicile becomes difficult because the insects must walk for feeding [56]. Centroid size was used in this research as a measure of overall head and wing size differences among *T. infestans* populations. Because of these high temperatures in boreal Chaco (Huacaya/Imbochi) (temperature higher than 30 °C and humidity lower than 20%), the specimens were smaller compared to those from the valley. Vilaseca et al. [12] similarly observed that the centroid size of *T. infestans* was larger in populations from the inter-Andean valleys compared to specimens from Chaco. In Chaco, variations were observed in *T. infestans* heads; males had a smaller head and a different shape compared to female heads. According to Hernández et al. [48], changes in the males’ head morphology have a dispersal genetic component. The variation in size among populations suggests strong differential selection and sensitivity to changes in environmental conditions [57,58,59,60,61]. Reproductive studies on insects have shown that the biological cycles associated with high temperatures would be shorter; therefore, the specimens tend to be smaller [8,9,53,62,63,64,65]. In contrast to size, analyses of organismal shape, which was proofed to be influenced by allometry, provide more reliable information about the phenotypic variation of populations representing high and stable heritability [66,67]. Although *T. infestans* head and wing shape comparisons revealed some differences between populations, clear site-specific population differentiation was not found. However, an environmental pattern in head and wing shape variation was detected when populations were pooled by sex (as already described in part A).

(C) Nutrition profile has been observed in variability in this study. Unfavorable environmental conditions lead to circumstances in which females have the priority to feed, therefore resulting in smaller males (in shape and size). Consequently, the females become bigger than the males as an adaptation mechanism to the environmental pressures in order to enhance fertility [53,54,55]. Males carry out the re-infestation process, so they tend to stay in the peridomicile (Chicken coops and pens) [21]. *T. infestans* mostly feed on bird blood. According to Natero et al. [68] and Lunardi et al. [69], when triatomines feed on bird blood, no variability in the head shape will occur. In this research, no dimorphism was observed in the shape of the head because both females and males fed on bird blood. When analyzing centroid size, females’ heads occur longer than male heads. Except for the intensity of feeding, the type of blood host also could have an influence on morphological variation in this species. According to Natero et al. [68], when *T. infestans* feeds on mammalian blood, a morphological widening and shortening effect of the head is observed, in contrast to individuals feeding on bird blood. Lunardi et al. [69] found phenotypic plasticity in *Triatoma williami* based on feeding on a mammal or bird blood as an adaptation process to the host. In this research, *T. infestans* from Chaco was found in the intra and peridomicile, and the males tend to fly from the peridomestic to intradomicile and feed on human/mammal blood, which evidently had a direct influence on the variability in size and shape of the male heads comparing with female heads.

At the population level, the variation of the first three principal components showed that there was no clear-cut separation between the populations, which is in agreement with a high proportion of misassignments and the findings of low population structure and no isolation by distance (using mitochondrial cytochrome b gene and microsatellite loci), as demonstrated by Giordano et al. [4] and Marcet et al. [70]. According to these authors’ findings, *T. infestans* populations are genetically similar. The slight wing shape differences detected (especially between wings) may be the result of emerging phenotypic plasticity. Phenotypic plasticity is often defined as the change in the phenotypic expression of a genotype in response to environmental factors [71] and has been shown to have significant evolutionary consequences [71,72].

## 5. Conclusions

The morphological variation of *T. infestans* from two different environments has been determined. The inter-Andean and Chaco populations showed the sexual size and shape dimorphism in relation to environmental factors and nutrition. The results of the present survey will serve as a starting point in further understanding the re-infestation processes to redesign the science-based vector control programs.

## Figures and Tables

**Table 1:** Pairwise comparison using Mahalanobis distance and Procrustes distance between females and males of *Triatoma infestans* inter-Andean Valley and Chaco population (* *p* < 0.0001).

View	Region	Mahalanobis Distance			Procrustes Distance		
		F/Chaco	F/Valley	M/Chaco	F/Chaco	F/Valley	M/Chaco
Head dorsal	F/Valley	1.81138 *			0.0123		
	M/Chaco	1.8254 *	2.2565 *		0.0132	0.0168 *	
	M/Valley	1.7989 *	1.3489	2.2707 *	0.0131	0.0073	0.0177*
Wings	F/Valley	2.5087 *			0.0235*		
	M/Chaco	1.3489	2.2660 *		0.0107	0.212	
	M/Valley	2.3459 *	1.5397 *	2.1843 *	0.0191	0.0187	0.0193

## Data Availability

The datasets used and/or analyzed during the current study are available from the corresponding author on reasonable request.
